# Identification of Cytotoxic T Lymphocyte Epitopes on Swine Viruses: Multi-Epitope Design for Universal T Cell Vaccine

**DOI:** 10.1371/journal.pone.0084443

**Published:** 2013-12-17

**Authors:** Yu-Chieh Liao, Hsin-Hung Lin, Chieh-Hua Lin, Wen-Bin Chung

**Affiliations:** 1 Division of Biostatistics and Bioinformatics, Institute of Population Health Sciences, National Health Research Institutes, Miaoli, Taiwan; 2 Institute of Bioinformatics and Structural Biology, National Tsing Hua University, Hsinchu, Taiwan; 3 Department of Veterinary Medicine, National Pingtung University of Science and Technology, Pingtung, Taiwan; Virginia Polytechnic Institute and State University, United States of America

## Abstract

Classical swine fever (CSF), foot-and-mouth disease (FMD) and porcine reproductive and respiratory syndrome (PRRS) are the primary diseases affecting the pig industry globally. Vaccine induced CD8^+^ T cell-mediated immune response might be long-lived and cross-serotype and thus deserve further attention. Although large panels of synthetic overlapping peptides spanning the entire length of the polyproteins of a virus facilitate the detection of cytotoxic T lymphocyte (CTL) epitopes, it is an exceedingly costly and cumbersome approach. Alternatively, computational predictions have been proven to be of satisfactory accuracy and are easily performed. Such a method enables the systematic identification of genome-wide CTL epitopes by incorporating epitope prediction tools in analyzing large numbers of viral sequences. In this study, we have implemented an integrated bioinformatics pipeline for the identification of CTL epitopes of swine viruses including the CSF virus (CSFV), FMD virus (FMDV) and PRRS virus (PRRSV) and assembled these epitopes on a web resource to facilitate vaccine design. Identification of epitopes for cross protections to different subtypes of virus are also reported in this study and may be useful for the development of a universal vaccine against such viral infections among the swine population. The CTL epitopes identified in this study have been evaluated *in silico* and possibly provide more and wider protection in compared to traditional single-reference vaccine design. The web resource is free and open to all users through http://sb.nhri.org.tw/ICES.

## Introduction

 Classical swine fever virus (CSFV), foot-and-mouth disease virus (FMDV) and porcine reproductive and respiratory syndrome virus (PRRSV) are debilitating pathogens in the swine industry, resulting in serious economic losses year-after-year. The development of effective vaccines against these pathogens is therefore of practical significance in the swine industry. Although neutralizing antibodies induced upon vaccination are highly effective in controlling disease and viral transmission, they do not confer cross-subtype protection and might become ineffective due to antigenic changes [1]. Currently, cellular immune responses, especially production of cytotoxic T lymphocytes (CTL), are receiving much attention due to their potential in developing efficient and cross-protective peptide vaccines against various viruses [2]. For example, the CTL epitope peptides could be used for the development of cross-protective human influenza vaccines, including recombinant viral vector and peptide vaccine [3-5]; the CTL epitope peptide identified for FMDV serotype O was cross-reactive to other FMDV serotypes [6]. However, most of the analyses were restricted to specific viral proteins and were only able to identify few CTL epitopes.

 A systematic approach based on the synthesis and evaluation of large sets of overlapping peptides has been proposed and used in screening CTL epitopes of viral proteins [6-12], however, it is a labor-intensive and time-consuming procedure. In Graham et al.'s study, a proteome-wide peptide library to screen T cell epitopes for classical swine fever virus was constructed; however, the group selected one reference sequence of a related virus, bovine viral diarrhoea virus (BVDV), to design the synthetic overlapping peptides spanning the entire length of the polyproteins from the virus with 16-mer peptides offset by four amino acid residues [11]. In other words, sequence variation, a common phenomenon in viral evolution, was not considered in this study. Such a flawed experimental design might produce inadequate memory T cells in recognition of classical swine fever viruses. Highly conserved internal antigens (nucleoprotein (NP) and matrix protein 1 (M1)) have been encoded in a Modified Vaccinia virus Ankara vector to increase T-cell responses and to provide longer-lasting protection against multiple influenza subtypes [5,13,14]. In addition, Goodman et al. have developed a universal T cell vaccine against influenza virus based on multi-epitope recombinant vaccinia virus containing epitopes of M1, NS1, NP, PB1 and PA proteins [3]. These influenza virus-related studies demonstrate the feasibility of universal T cell vaccine design based on multiple epitopes and the demand of effective CTL epitopes. In contrast to peptide library screen, computational analyses have been proposed to perform efficient and systematic screening for CTL epitopes and successfully applied to human viruses, such as dengue virus, human respiratory syncytial virus and human influenza virus [4,15-17]. Although great progress has been made in NetMHCpan [18], there is still limited knowledge on the haplotypes of swine leukocyte antigens (SLA) in swine. The gaps in knowledge might thus limit the application of the NetMHCpan to the identification of SLA haplotype-specific CTL epitopes.

 In this study, we have integrated a bioinformatics pipeline to analyze swine viral sequences in order to resolve the above-mentioned challenges: (1) genetic variation, (2) incomplete screening from particular surface proteins, and (3) inappropriate prediction based on non-swine leukocyte antigens. We thus have designed and constructed a web resource, called identification of cytotoxic T lymphocyte epitopes for swine viruses (ICES) for the identification of CTL epitopes of swine viruses such as CSFV, FMDV and PRRSV. Predictions of proteasomal cleavage sites and binding affinity to SLA as well as calculations of sequence conservation were implemented in ICES. Users are able to freely access ICES online via http://sb.nhri.org.tw/ICES for genome-wide scan and epitope search for potential CTL epitopes of swine viruses. In addition, we have demonstrated that experimentally-validated epitopes can be found in support the predictions of ICES. Taken together, ICES is a valuable resource for the design of effective peptide vaccines for swine viruses.

## Materials and Methods

### Sequence collection and peptide analysis

**Figure 1:**
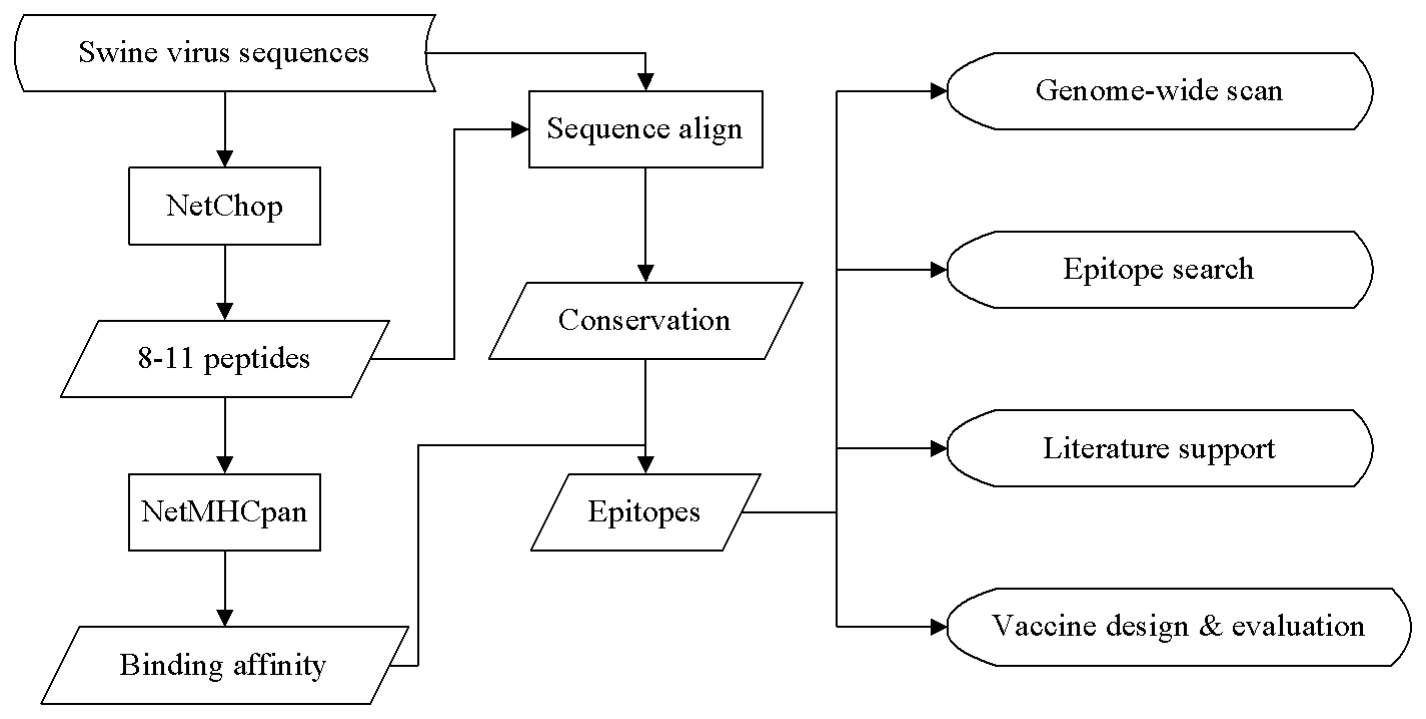
A schematic overview of identification of cytotoxic T lymphocyte epitopes for swine viruses (ICES).

 The coding sequences of swine viruses including CSFV, FMDV and PRRSV were retrieved from NCBI GenBank [19]. For each subtype (genotype or serotype) of swine virus, a reference strain with complete genome was selected. Each coding sequence was assigned to its corresponding subtype based on sequence similarity to the reference strain if the information regarding genotype or serotype was absent. A stand-alone NetChop 3.1 was utilized to predict proteasomal cleavage sites of all the swine viral sequences [20]. Possible cleavage sites were generated by using both C-term and 20S methods implemented in NetChop with thresholds of 0.5 and 0.6, respectively. To limit the sequence to a small linear peptide with a fixed length, the cleavage sites of each sequence was scanned to generate all possible linear peptides in the range of 8- to 11-amino acid residues. Those peptides were subsequently utilized to predict their binding affinities to swine leukocyte antigens (SLA) via a stand-alone NetMHCpan 2.4 [18]. Forty-five SLA alleles provided in the NetMHCpan were all selected for peptide-binding prediction. Furthermore, each peptide was aligned to its reference genome to establish its location and to calculate its sequence conservation. A schematic overview is shown in Figure 1.

### Web implementation

 We have performed the viral sequence analyses, including the predictions of proteasomal cleavage sites and binding affinity as well as the calculation of sequence conservation for peptides, and have stored all data in MySQL database. We have constructed ICES with JSP and JavaScript and constructed it on a Linux machine. The server is free and open to all users via http://sb.nhri.org.tw/ICES. Four schemes: genome-wide scan, epitope search, literature support, and vaccine design & evaluation were implemented in ICES for the identification and evaluation of CTL epitopes of swine viruses.

### *In silico* evaluation

 We have identified genome-wide CTL epitopes for CSFV, FMDV and PRRSV (e.g., Table 1 for FMDV type O viruses). We used FMDV as an example for *in silico* evaluation. We hypothesized that a tailored viral vector expressing specific CTL epitopes is able to induce T-cell immunity, and the pre-existing immunity could be activated when exposed to the corresponding antigens. We therefore assumed that the as-designed memory T-cell immunity could recognize and clean viruses containing the specific epitopes. We evaluated vaccine efficacy by means of counting the number of peptides in an examined virus correspond with the as-designed epitopes. Viruses of whole genome sequence (≥ 10) were tested. 41 Type Asia 1, 81 Type A, 16 Type C and 138 Type O FMDV viral sequences were thus used for evaluation. We firstly segmented the genome polyproteins into peptides using NetChop prediction [20]. Subsequently, we examined whether the peptides corresponded to the as-predicted CTL epitopes. For each virus under examination, we summarized the numbers of matched peptides and the corresponding SLA. For example, the polyprotein of a reference strain of FMDV type O virus AF308157 (protein accession: AAG45408) was divided into 2309 peptides of length 8-11 by NetChop. Among the 2309 peptides, 19 peptides correspond with the CTL epitopes shown in Table 1 and these peptides were predicted to be strongly bound to 29 various SLAs.

**Table 1:** CTL epitopes of FMDV type O viruses predicted by using genome-wide scan.

CTL epitopes predicted	Position	Protein	Alleles	Affinity^#^	Rank	Conservation
EPFFDWVY	65	L	SLA-1*0701-0702	Null	0.08	0.92
YMQQYQNSM	227	VP4	SLA-1*1101, SLA-3*0302	Null	0.01-0.08	1
SSVGVTYGY	314	VP2	SLA-2*0302, SLA-2*1002	40.25	0.05	0.97
RFFKTHLF	346	VP2	SLA-3*0602, SLA-3*0701	Null	0.01-0.08	0.99
AYMRNGWDVEV	385	VP2	SLA-2*0701	Null	0.08	0.98
RELYQLTL	421	VP2	SLA-3*0501-0503	Null	0.05	0.93
YQLTLFPHQF	424	VP2	SLA-3*0302, 0501-0503, SLA-6*0101-0105	Null	0.03-0.05	0.97
KARYMIAY	622	VP3	SLA-3*0301, 0303, 0304, 0401, 0601	Null	0.05-0.08	0.9
IIATTNLY	1309	2C	SLA-1*0601	Null	0.08	1
FQYDCALL(NGM)	1369	2C	SLA-2*1001, SLA-3*0302	36.43-62.01	0.03-0.08	0.97
MLSDAALMVL	1720	3C	SLA-2*0201-0202	242.31	0.05	0.99
WQRFGTHFAQY	2075	3D	SLA-3*0301-0304	Null	0.03-0.05	0.99
AQYRNVWDVDY	2083	3D	SLA-3*0601	Null	0.08	0.9
NTILNNIYV(LY)	2159	3D	SLA-2*0502, SLA-2*0101	105.69	0.08	0.98
SITDVTFLK	2231	3D	SLA-1*1201	Null	0.05	1
HMDYGTGFY	2243	3D	SLA-1*0601, SLA-2*0102	57.98	0.05-0.08	0.99
KTLEAILSF	2258	3D	SLA-1*0501, SLA-1*1301	Null	0.05-0.08	0.99
FEPFQGLF(EI)	2295	3D	SLA-6*0101-0105	Null	0.08	1
FEIPSYRSLY	2302	3D	SLA-2*0402, SLA-3*0302	Null	0.08	0.99

^#^ Affinity (IC_50_ value in nM) is assigned "Null" if quantitative binding data not available for the swine alleles [18].

## Results

A schematic overview of the integrated bioinformatics pipeline depicted in Figure 1 was proposed to construct a web resource, named ICES, for the identification of the CTL epitopes of swine viruses in this study. Available coding sequences of CSFV, FMDV, and PRRSV were all retrieved from NCBI GenBank. The proteasomal cleavage sites for the aforementioned viruses were predicted using NetChop. The predictions of the binding affinity of 8-11 amino acid-long linear peptides were subsequently generated by using NetMHCpan. The position of the first amino acid of the peptides relative to the reference strain was recorded; with this information in hand, the sequence conservation of each peptide was subsequently calculated. With the predicted CTL epitopes of swine viruses available, ICES was equipped with four main schemes: genome-wide scan, epitope search, literature support and vaccine design & evaluation.

**Figure 2:**
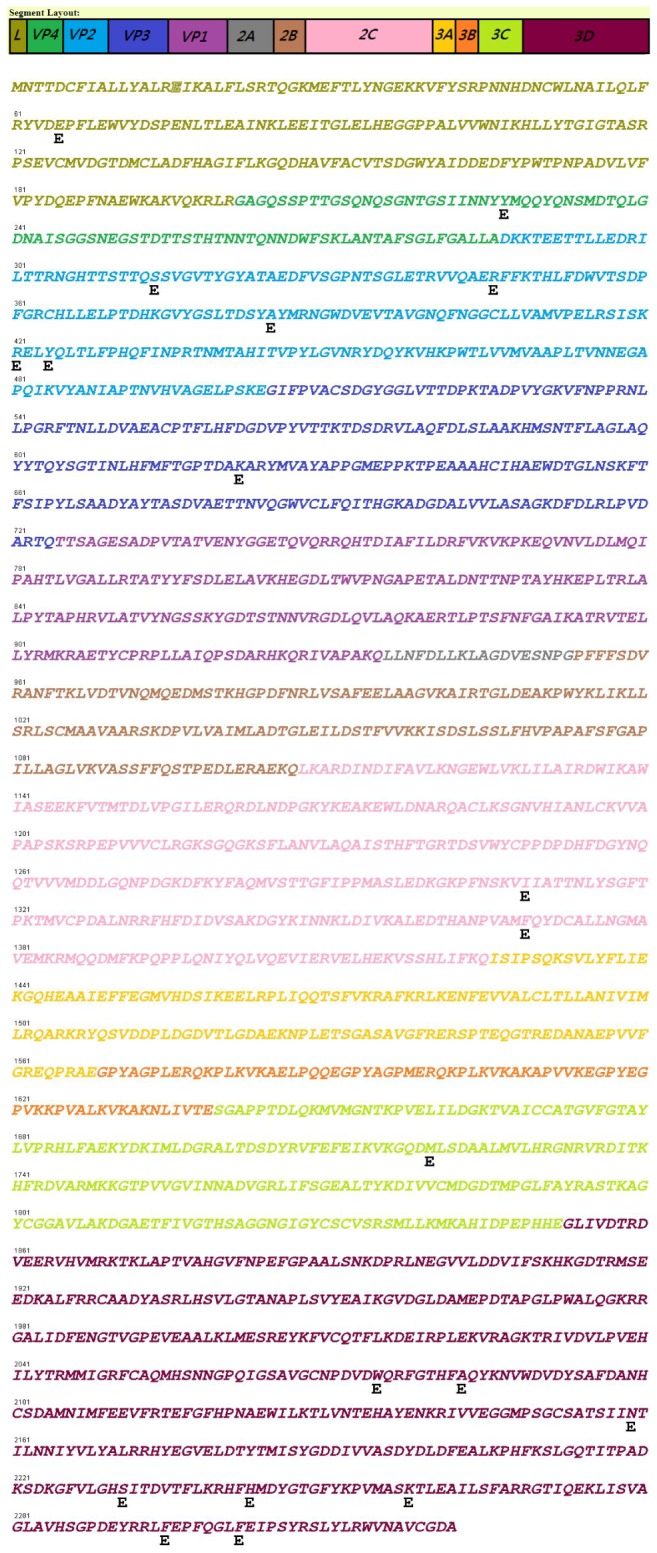
A genome-wide scan for CTL epitopes of FMDV type O viruses generated via the identification of cytotoxic T lymphocyte epitopes for swine viruses (ICES) with the threshold of sequence conservation ≥ 0.9 and strong binding affinity (affinity IC_50_ ≤ 50 nM or rank among the top 0.1%).

In the paging of genome-wide scans, users are able to select a virus and its subtype for the identification of possible CTL epitopes with a different parameter setting. For example, a result of the genome-wide scan for FMDV type-O virus is output by ICES (Figure 2), exhibiting the starting sites of predicted CTL epitopes, denoted as "E" below the reference genome sequence (GenBank accession no. AF308157). Furthermore, users are able to obtain the information of binding affinity and sequence conservation for the predicted peptide of the CTL epitope by clicking on "E" in the interactive interface of ICES. Accordingly, a list of genome-wide scans for CTL epitope prediction of FMDV type-O virus is summarized in Table 1. Twenty-two peptides of strong binding affinity (affinity IC_50_ ≤ 50 nM or rank among the top 0.1%) located in the nineteen positions across the reference genome of FMDV type-O virus (AF308157, as shown in Table 2) were identified and confirmed to be highly conserved (sequence conservation ≥ 0.9). Those peptides with strong binding affinity to the various alleles of swine leukocyte antigen (SLA) are expected to be the proper peptide antigens for the development of vaccines against FMDV type-O viruses. ICES could, therefore, be employed to discover potential CTL epitopes for the various subtypes of swine viruses.

**Table 2:** A summary of CTL epitope predictions for swine viruses.

**Virus**	**Genotype/ Serotype**	**Ref. strain (accession no.)**	**No. of viruses**	**Predicted epitopes (position)^#^**
FMDV	Asia 1	AY304994	431	SSVGVTYGY (314), FQYDCALLNGM (1367)
	Sat1	AY593838	271	None
	Sat2	AY593849	336	None
	Sat3	AY593850	70	None
	Type A	AY593751	991	SSVGVTYGY (314), FQYDCALLNGM (1369)
	Type C	AF274010	114	None
	Type O	AF308157	1711	SSVGVTYGY (314), FQYDCALLNGM (1369)

PRRSV	Type 1	AY366525	1443	VSYYLTLY (3545)
	Type 2	AY150564	12012	SQHGLTLPL (1640), RMMGHAWTPL (2030), FTWYQLASY (3811), YQLASYASY(I) (3814), YLASRLPM (4121)

CSFV	Type 1	X87939	845	SEFLLLSLV (549), NSASTTAFLI (655), VVYFLLLY (1085), FTMWADILTLI (1288), REMNYDWSL (2140), AVAFSFLLMY (3724)
	Type 2	HQ148063	68	SEFLLLSLV(I) (549), NSASTTAFLI (655), REMNYDWSL (2140), AVAFSFLLMY (3724)

^#^ Prediction scores obtained from NetMHCpan are stronger than 50 nM and among the top 0.1% rank and sequence conservation is greater than or equal to 90%.

With respect to the scheme of epitope search, we have designed three query interfaces for cross-virus epitopes, cross-subtype epitopes as well as binding affinity and conservation. However, no peptide could be identified as a cross-virus epitope for the three swine viruses; we removed this query interface accordingly. The cross-subtype epitopes could be identified via an epitope search in ICES. As shown in Table 2, four peptides (SEFLLLSLV, NSASTTAFLI, REMNYDWSL and AVAFSFLLMY) are the cross-genotype epitopes for CSFV. In addition, ICES has predicted two peptides, SSVGVTYGY and FQYDCALLNGM, common among the unique serotypes of the FMD virus. These two peptides are likely to induce cytotoxic T cells, albeit further experiments are required for verification. It should be noted that the binding affinity for the predicted epitopes was limited to an affinity of ≤ 50 nM and rank among the top 0.1%, which is more stringent than the rule established in the Table 1. Such strict rules could assure peptide candidates for successful vaccine design. The query interface for binding affinity and conservation was employed to search for the binding affinity and sequence conservation of the peptide. Users are able to input their desired peptide and get the corresponding information from the ICES.

Data achieved, in previous studies [1,21,22], by applying NetMHCpan peptide prediction algorithm to FMDV indicated that a considerable amount of peptides predicted by the NetMHCpan were strongly bound to swine leukocyte antigens. For example, the peptide MTAHITVPY predicted to be bound to SLA-1*0401 and SLA-2*0401 in <0.1% rank score was actually bound to the SLA-1*0401 and SLA-2*0401 major histocompatibility complex class I proteins [22]. Patch et al. used recombinant human adenovirus vectors to deliver FMDV capsid antigens and found such a means of vaccination could enhance CTL response. However, they also found that the peptide MTAHITVPY less displayed in the complex of MHC molecules of the infected cells because the capsid proteins were cleaved differently by the proteasome [1]. In our study, proteasomal cleavage has been taken into consideration by implementing NetChop prediction into ICES, and thus the peptide MTAHITVPY was not predicted as CTL epitope by ICES. Except those peptides bound with the restricted SLAs [1,21,22], few studies have identified FMDV CTL epitopes [23]. Since all the epitopes identified in the ICES are *in silico* predictions, further *in vitro* and *in vivo* experimental verification is required. To address the issue of reliability, we have surveyed related literature in support of the CTL epitope predictions (Table 3). In the case of FMDV, Gao et al. identified two peptides RRQHTDVSF and RTLPTSFNY bound with the reconstructed SLA-2 protein [23], and both peptides were successfully predicted strong binding with restricted SLA-2 alleles in our system. Similar evidences can also be generally found for PRRSV and CSFV, as listed in Table 3. The three peptides, CLFAILLAT, CAFAAFVCFVIR and KPEKPHFPL, found in Diaz et al's study, were firstly predicted using human or cattle alleles and then evaluated *ex vivo* [24], which may not be effective CTL epitopes of PRRSV. Therefore, we believe that most of the experimentally-corroborated CTL epitopes can support the CTL epitope predictions for swine viruses provided by ICES. On the other hand, equipped with the functionality of a genome-wide scan, ICES could be used to identify CTL epitopes with strong binding affinities and high sequence conservation for swine viruses to facilitate vaccine design.

**Table 3:** Supporting evidences of CTL epitope predictions.

**Epitope**	**Subtype**	**Position**	**Note^*#*^**	**Reference**
FMDV (foot-and-mouth disease viruses)				
RRQHTDVSF	Type O	750	Highly supportive	[23]
RTLPTSFNY	Type O	881	Highly supportive	[23]
PRRSV (porcine reproductive and respiratory syndrome viruses)				
CLFAILLAT	Type 1	4596	Not supportive	[24]
CAFAAFVCFVIR	Type 1	4721	Not supportive	[24]
KPEKPHFPL	Type 1	5028	Not supportive	[24]
FMLPVAHTV	Type 1	5083	Highly supportive	[24]
TMPPGFELY	Type 2	2702	Highly supportive	[28]
LAALICFVIRLAKNC	Type 2	4765	Supportive	[8]
KGRLYRWRSPVII/VEK	Type 2	4797	Supportive	[8]
CSFV (classical swine fever viruses)				
KHKVRNEVMVHWFDD	Type 1	1446	Weakly supportive	[10,26,27]
ENALLVALF	Type 1	2276	Supportive	[12,25]

^***#***^ Highly supportive: peptide satisfies peptide identity and strong affinity prediction (affinity IC_50_ ≤ 50 nM or rank among the top 0.1%)

Supportive: peptide satisfies peptide identity and weak binding affinity prediction (affinity IC_50_ ≤ 500 nM or rank among the top 1%) Weakly supportive: peptide partly satisfies peptide identity and weak affinity prediction

**Figure 3:**
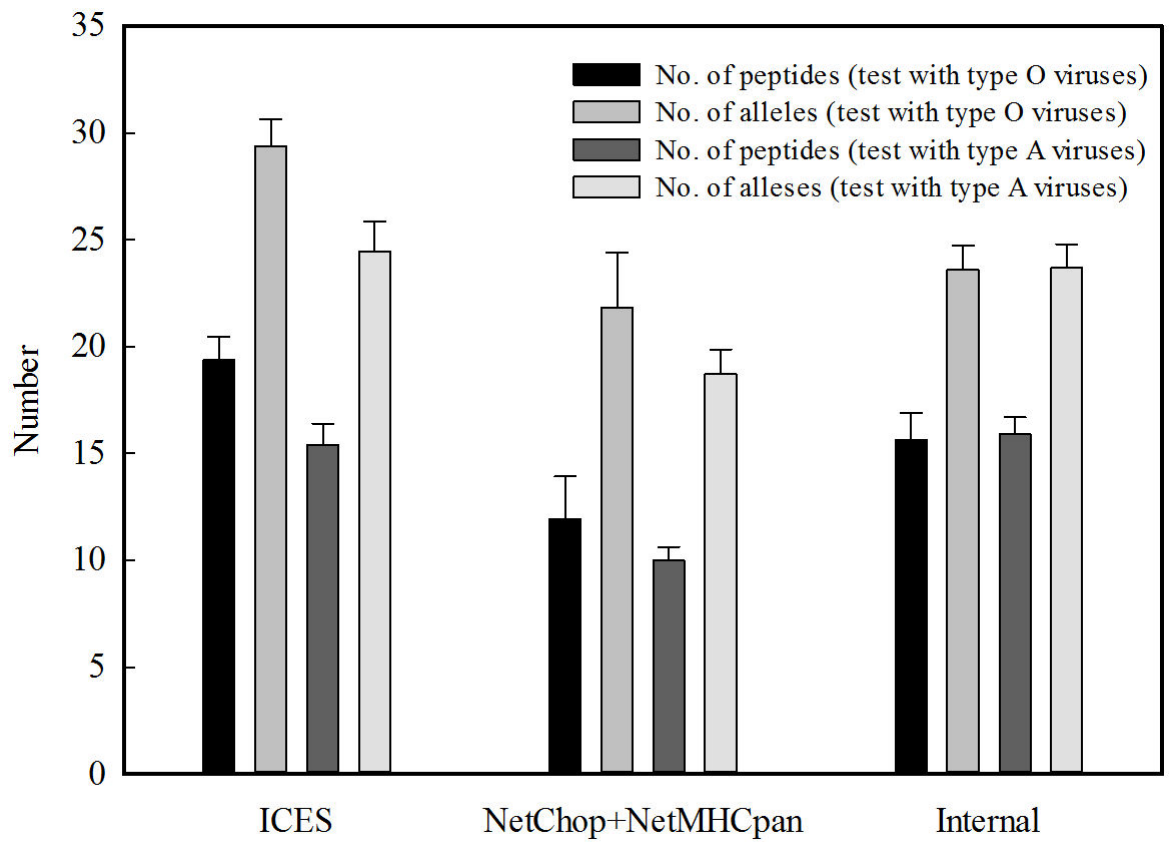
*In silico* evaluation on multiple CTL epitopes identified by various methods.

**Figure 4:**
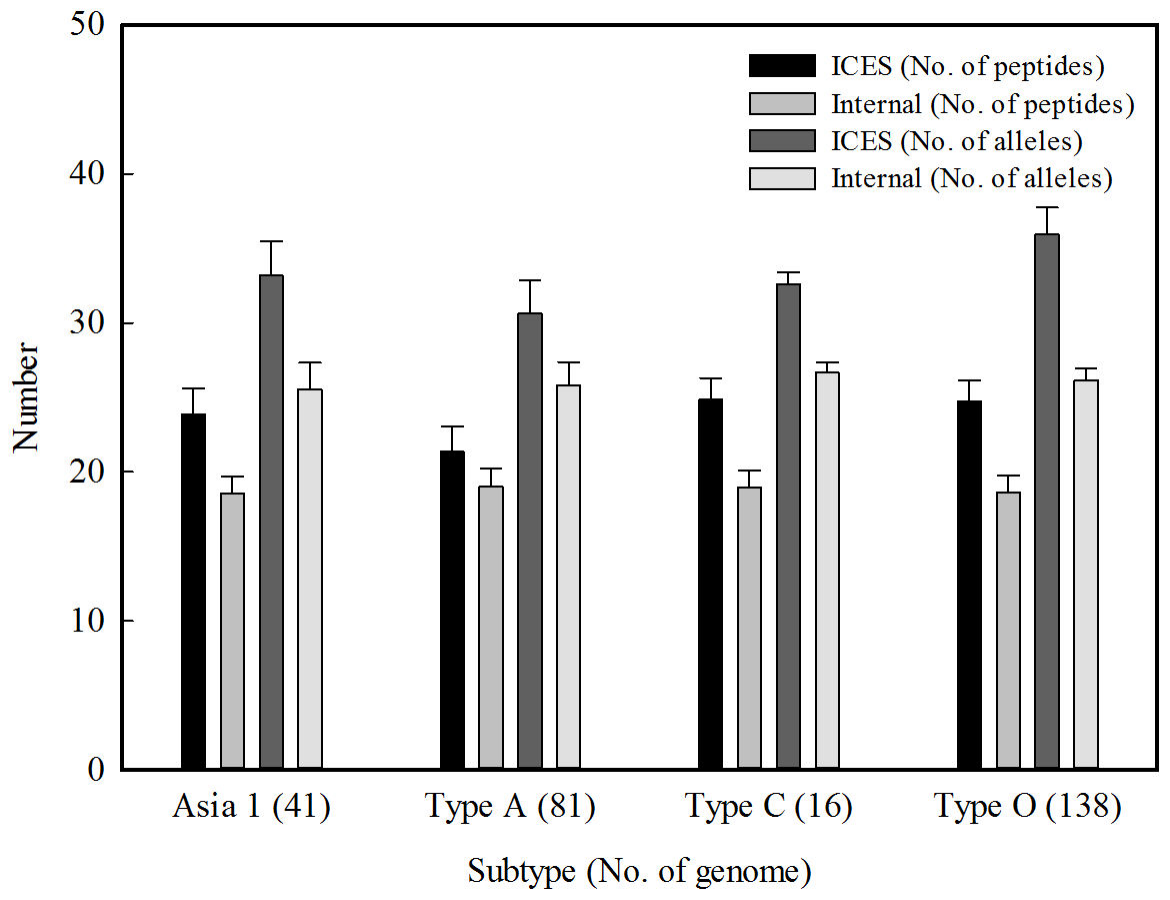
*In silico* evaluation on cross-subtypic CTL epitopes.

The aim of this study is to assist in vaccine design. Since multiple CTL epitopes can be incorporated in a non-disease-causing viral vector for development of universal T cell vaccine, we have identified 47, 36 and 77 CTL epitopes for heterosubtypic CSFV, FMDV and PRRSV, respectively (the list is shown in the paging of vaccine design & evaluation of ICES). *In silico* evaluation on multiple-epitope design for CTL vaccine was carried out in this study. In addition to the 22 peptides yield by genome scan function of ICES (Table 1), we separately conducted NetChop and NetMHCpan for a reference strain of FMDV type O viruses (AF308157, protein accession: AAG45408) to find 42 CTL epitopes. Among the 42 peptides, 20 peptides located in internal proteins were also used for further analysis. Based on the hypothesis that a tailored viral vector expressing specific CTL epitopes is able to induce T-cell immunity, and the pre-existing immunity could be activated when exposed to the corresponding antigens. We thus evaluated the vaccine efficacy by counting the number of peptides in an examined virus correspond with the as-designed epitopes. To equally compare our epitope predictions with the epitopes obtained from a single reference, 20 epitopes were randomly selected from the 22 epitopes (ICES) and the 42 epitopes (NetChop+NetMHCpan), respectively. The number of matched peptides and the number of the corresponding alleles, observed in Figure 3, are significantly (t-test, p-value <10^-5^) higher than that obtained from a single-reference design by NetChop+NetMHCpan, in testing type A and type O viruses. Although, in testing type A viruses, the numbers of peptides and alleles obtained from internal peptides are close to that from ICES, they are only 80% of the numbers obtained from ICES in testing type O viruses. In order to design cross-subtypic vaccine for FMDV viruses, we have identified 36 CTL epitopes from ICES based on all collected viral sequences and 38 internal CTL epitopes based on the 7 single reference strains (FMDV viruses in Table 2), respectively, and further evaluated their efficacy in recognizing various subtypes. As can be observed in Figure 4, the ICES-designed epitopes provide more (in terms of the number of peptides) and wider (in terms of the number of alleles) protection than the internal protein-designed peptides (t-test, p-value <10^-5^). On the whole, ICES is a valuable resource of potential CTL epitopes for swine vaccine design.

## Discussion

 CSF, FMD and PRRS are considered key challenges in the swine industry. A number of studies have been investigated for the identification of CTL epitopes of CSFV [10,12,25-27], FMDV [1,21-23] and PRRSV [8,24,28]. Most of these investigations have focused on specific structural proteins, e.g. GP4 and GP5 of PRRSV, VP1 of FMDV and E2 of CSFV. However, unlike antibodies, CTL epitopes can be identified from any protein constituent of the virus, whether they can be found internally or on its surface. Therefore, a genome-wide scan for CTL epitopes of the swine virus is warranted. Although overlapping peptide libraries have been designed to detect T-cell epitopes [8,29], it is not practical to conduct a complete peptide design that spans the entire polyproteins of the virus as it is a very labor-intensive and time-consuming process. Even though Diaz et al. [24] has proposed the *in silico* prediction of T-cell epitopes of PRRSV, they neither searched for the whole protein nor utilized alleles of SLA, which might lead to incomplete and/or false positive identification of CTL epitopes. The established integrated bioinformatics pipeline was hence designed to facilitate the genome-wide identification of CTL epitopes for swine viruses.

 Many CTL epitope prediction tools are publicly available [30-33]. Nevertheless, few tools provide peptide binding data specific to swine alleles. In Pan et al.'s study, SLA was shown to exhibit only 72.7% and 66.2% sequence identity to human and mouse class I major histocompatibility complexes (MHC) [28], respectively, which clearly suggests that CTL predictions for swine viruses based on human and mouse alleles are inappropriate. Since NetMHCpan utilized porcine MHC class I molecules for CTL epitope predictions [18,21], it was incorporated into the pipeline. In addition to peptide binding affinity to MHC class I, protein degradation instigated by the proteasome and peptides transportation to the endoplasmatic reticulum (ER) by transporters associated with antigen presentation (TAP) molecules are also important to MHC class I presentation. NetCTLpan was therefore implemented to integrate predictions of proteasomal cleavage, TAP transport efficiency and MHC class I binding affinity for human CTL epitope predictions in consideration of all the three issues [34]. As described in the NetChop server, proteasome structure is conserved and is able to produce reliable predictions for other mammalian proteasomes; NetChop was thus applied to predict proteasomal cleavage sites for swine viruses. However, owing to the lack of supporting evidence showing that human TAP transport efficiency is similar to that in swine cases as well as the extremely low weight on TAP transport efficiency in NetCTLpan [34], NetChop and NetMHCpan were thus integrated into the ICES (Figure 1).

 We have analyzed all the viral sequences of CSFV, FMDV and PRRSV that were downloaded from GenBank in order to generate a genome-wide list of potential CTL epitopes in the ICES. As shown in Table 1, among the 22 predicted CTL epitopes for FMDV type-O virus, seven epitopes were located in structural proteins (none in VP1) while the others occupied the non-structural protein segment, indicating that the search for CTL epitopes within the surface protein domain, e.g. VP1 of FMDV, would lead to inconclusive CTL epitope identification or the discovery of peptides with low sequence conservation. For example, Gao et al. has found two 9-mer peptides from the FMDV VP1 region (RRQHTDVSF and RTLPTSFNY in Table 3), which are able to bind with SLA-2 [23], consistent with the binding affinity predictions (RQHTDVSFIL and RTLPTSFNY are among the top 0.1% rank, the details can be seen in the website of ICES). However, the peptides are not conserved (sequence conservation ranges from 0.53 to 0.57) such that the as-prepared vaccines might provide insufficient protection against FMDV type-O viruses. On the other hand, vaccine design based on internal proteins of single reference strain might lead to the reduction in the broadness of restricted SLA, e.g. the peptide YQLTLFPHQF predicted strong bound with 9 restricted SLAs but was not found by the internal protein approach. Similar results are observed in Figure 4. Therefore, we collected as many swine viral sequences as possible and analyzed their conservation, along with the prediction of binding affinity in order to screen peptide candidates for the rational vaccine design. Vaccine inducing CD8^+^ T cell-mediated immune responses might be long-lived and cross-serotype and thus deserves further attention [6]. ICES was therefore employed to identify suitable peptide candidates for validation (Table 2). With this widely-accessible web resource, researchers are freely able to locate CTL epitopes and further design multiple-epitope vaccine for validation. Instead of identifying and validating a specific CTL epitope for a particular scenario, we have proposed and developed an integrated bioinformatics pipeline to reduce labored efforts of the virus researcher while increasing the effectiveness of the vaccine formulation. ICES is currently the only web resource to provide a thorough scan of swine viruses for CTL epitope identification. Furthermore, epitope-based peptide vaccines can offer several advantages over the vaccines with whole antigens. The potential benefits include the precise designing of the most important antigens with highly immunogenic and conserved epitopes, relatively easy to construct and to produce, as well as the absence of infectious potential.

 In summary, a systematic bioinformatics pipeline has been designed to identify potential CTL epitopes of CSFV, FMDV and PRRSV; the as-predicted CTL epitopes were subsequently integrated into ICES. Therefore, ICES has been demonstrated as a valuable resource for swine vaccine design. In addition, the framework of ICES can easily be adapted for use among other emerging human pathogens, such as dengue virus and HIV, and thus making a great impact on the control of infectious diseases.
